# Tracking defined microbial communities by multicolor flow cytometry reveals tradeoffs between productivity and diversity

**DOI:** 10.3389/fmicb.2022.910390

**Published:** 2023-01-05

**Authors:** Firas S. Midani, Lawrence A. David

**Affiliations:** ^1^Center for Genomic and Computational Biology, Duke University, Durham, NC, United States; ^2^Department of Molecular Virology and Microbiology, Baylor College of Medicine, Houston, TX, United States; ^3^Department of Molecular Genetics and Microbiology, Duke University, Durham, NC, United States

**Keywords:** *Bacteroides*, cross feeding, flow cytometry, carbon sources, microbial ecology

## Abstract

Cross feeding between microbes is ubiquitous, but its impact on the diversity and productivity of microbial communities is incompletely understood. A reductionist approach using simple microbial communities has the potential to detect cross feeding interactions and their impact on ecosystem properties. However, quantifying abundance of more than two microbes in a community in a high throughput fashion requires rapid, inexpensive assays. Here, we show that multicolor flow cytometry combined with a machine learning-based classifier can rapidly quantify species abundances in simple, synthetic microbial communities. Our approach measures community structure over time and detects the exchange of metabolites in a four-member community of fluorescent *Bacteroides* species. Notably, we quantified species abundances in co-cultures and detected evidence of cooperation in polysaccharide processing and competition for monosaccharide utilization. We also observed that co-culturing on simple sugars, but not complex sugars, reduced microbial productivity, although less productive communities maintained higher community diversity. In summary, our multicolor flow cytometric approach presents an economical, tractable model system for microbial ecology using well-studied human bacteria. It can be extended to include additional species, evaluate more complex environments, and assay response of communities to a variety of disturbances.

## Introduction

Microbes inhabit diverse habitats and play a critical role in biogeochemical cycles, industrial biotechnology, and the health of humans, animals, and plants. Deeper insight into the ecological rules governing microbial growth, assembly into communities, and response to environmental changes will facilitate the design and control of microbial communities that are productive, resilient, and beneficial to their habitats. Toward this end, bottom-up, reductionist approaches that examine the growth of individual or pairs of microbial species have been successfully pursued to elucidate some of those ecological rules (Coyte and Rakoff-Nahoum, 2019). In particular, pairwise interactions between microbial species have been comprehensively investigated and shown to predict higher-order microbial structure and dynamics (Friedman et al., 2017; Venturelli et al., 2018). Such interactions can involve resource competition, cross feeding, antibacterial defenses, interspecies signaling, and alterations of the local abiotic environment (Pierce and Dutton, 2022). Still, pairwise interactions are often context-dependent and may materialize only in the presence or absence of additional members in a community (Kelsic et al., 2015; Momeni et al., 2017; Sundarraman et al., 2020). It is therefore likely that higher-order (e.g., three- and four-way) interactions must be measured to predict or recapitulate overall community dynamics with fidelity. Indeed, it has been shown that pairwise interactions only predicted the composition of seven-member communities with an accuracy of 62.5% while incorporating observations of three-species outcomes improved composition prediction accuracy to 86% (Friedman et al., 2017). Similarly, modeling emergent functions of microbial communities such as colonization resistance of pathogens often requires a small consortium of three or more microbes (He et al., 2014; Wei et al., 2015; Hromada et al., 2021). Therefore, reductionist approaches that model the growth of multiple species in a community, and not simply pairs of microbes, are needed to better understand how microbes assemble, interact, and provide ecosystem services.

Several techniques can count multiple unique microbes in a defined community, but these methods are often limited by the tradeoffs between the size of microbial communities (number of unique microbes included), throughput (number of unique conditions assayed), taxonomic resolution, and cost. Standard microbiological approaches (counting colony-forming units; Friedman et al., 2017), fluorescent probe-based imaging (Mark Welch et al., 2016), and marker-specific quantitative polymerase chain reactions (qPCRs; Mee et al., 2014) remain viable approaches for studying microbial ecology but are challenging to scale. For example, assessment of several bacteria in a community with qPCR requires engineering primers with comparable amplification efficiency (Brankatschk et al., 2012). The number of qPCR assays also increases linearly with community size unless multiple primer-probe pairs are precisely designed for use in multiplex qPCR assays (Smith and Osborn, 2009). Further, amplicon sequencing such as 16S rRNA gene sequencing can characterize diverse microbial communities in a high-throughput fashion but lacks taxonomic resolution at the species or strain level (Johnson et al., 2019). Metagenomic sequencing that can distinguish species or strains can be costly which curtails its use in high-throughput screens of microbial interactions. Thus, novel approaches are needed to rapidly and economically quantify the absolute abundance of at least three unique species in a defined microbial community.

Flow cytometry has the potential to address the challenges of scaling bottom-up ecological modeling. In flow cytometry, cells flow through laser beams one at a time and respond by scattering light or emitting light at specific wavelengths based on their optical and fluorescence properties (Davey and Kell, 1996; Adan et al., 2017). Using appropriate chemical staining dyes, microbiologists have demonstrated that flow cytometry can detect many cellular phenotypes including cell viability, Gram status, or metabolic activity (Anvarian et al., 2016; Buysschaert et al., 2016; Duquenoy et al., 2020). Flow cytometry also can quantify the absolute abundance of a specific microbe in a community (Polak et al., 2019) or the absolute abundance of all microbes in aquatic, soil, or fecal samples (Props et al., 2017; Vandeputte et al., 2017; Khalili et al., 2019). By clustering cells based on their optical and fluorescence properties, cytometric fingerprinting can also quantify the absolute abundance of many different groups of microbes in a complex sample (Koch et al., 2013; Koch and Müller, 2018). Fingerprinting can also broadly trace the ecological structure, stability, and function of microbial communities over time (Liu et al., 2018). However, in this approach, the identity of each group remains unknown without *a priori* knowledge of the cytometric fingerprint of each group based on intrinsic autofluorescence properties or downstream sorting of cell clusters followed by standard molecular techniques (Jehmlich et al., 2010; Zimmermann et al., 2016). Thus, the lack of taxonomic resolution by multicolor flow cytometry has limited its adoption in microbial ecology.

Multicolor flow cytometry is, however, suitable for bottom-up ecological modeling that relies on a synthetic community of known, tractable microbes. Natural strain-level heterogeneity in morphology is sufficient to distinguish tens of closely related species (Buysschaert et al., 2018) and can be improved by incorporating supervised machine learning that maps cytometric fingerprints to defined species (Pereira and Ebecken, 2011; Rubbens et al., 2017; Ludwig et al., 2019; Özel Duygan et al., 2020). Strain-level heterogeneity can also be artificially introduced by engineering microbes with strain-specific fluorescent reporters (Tracy et al., 2010). By using multiple fluorescence reporters with minimal spectral overlap, multiple unique microbes can be tracked in a complex sample. For example, the absolute abundances of three yeast species, encoded with either a red, green, or blue fluorescence protein, can be quantified simultaneously using multicolor flow cytometry (Conacher et al., 2020). To date, however, multicolor flow cytometry has not been used in any published studies to track multiple species for the purpose of bottom-up ecological modeling.

We therefore aimed to develop an application of multicolor flow cytometry capable of distinguishing multiple, closely related gut microbes in high-throughput fashion to allow more robust, bottom-up ecological modeling. To test the utility of our approach in the study of microbial ecology, we focused on cross feeding as a case study. Cross feeding is a common ecological phenomenon in which microbes consume nutrients that are secreted or degraded by other members of the community (Kehe et al., 2021). As a model microbial community, we chose the *Bacteroides* genus, known in the human gut for its broad ability to degrade dietary fiber, facilitating a variety of cross feeding interactions (Wexler and Goodman, 2017; Goyal et al., 2018). Different *Bacteroides* species can share nutrients in a nonspecific or cooperative manner in which one species digests polysaccharides into smaller components that can be shared with other species even at a cost to itself (Rakoff-Nahoum et al., 2014, 2016; Huus et al., 2021; Murakami et al., 2021). Hence, we applied multicolor flow cytometry and machine learning to rapidly detect cross feeding interactions between four fluorescent *Bacteroides* species using monocultures and co-cultures on individual carbon substrates. We also tested whether resource competition or substrate complexity impacts the productivity and diversity of *Bacteroides* communities. Broadly, our approach provides a simple model system for microbial ecology using well-studied human microbes that can be expanded to include additional species, evaluate more complex environments, and assay response of communities to a variety of disturbances.

## Results

### Multicolor flow cytometry quantitatively distinguishes *Bacteroides* species

Our initial model system comprised six *Bacteroides* species with chromosomally integrated fluorescence reporters. These species expressed either one of three levels of green fluorescent protein (GFP) expression plus one of two levels of mCherry expression (Whitaker et al., 2017); see Supplementary Table 1 for description of strains. Confocal microscopy distinguished these species at the single cell level with high accuracy (Whitaker et al., 2017).

**Figure 1:**
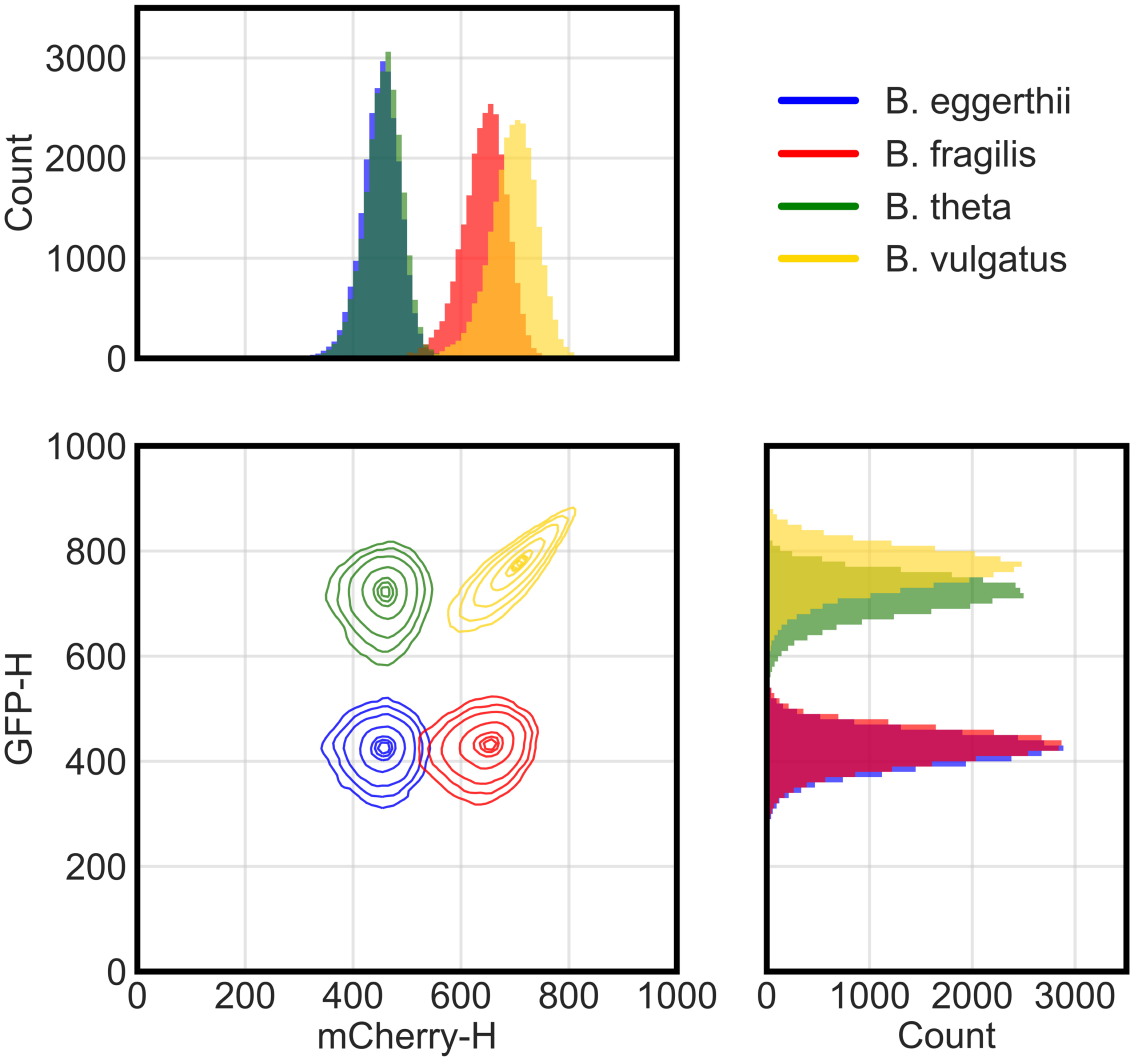
Flow cytometry distinguishes four fluorescent Bacteroides species. Flow cytometry analysis of cellular events (total of 25,000 events per species) from four species pooled together post-processing. Species were grown overnight in rich media then washed and incubated in cold aerobic conditions for approximately 12 h prior to analysis with flow cytometry. Cellular events were characterized by height of their signals in either green (GFP-H) or red (mCherry-H) fluorescence channels. The two-dimensional panel shows contour lines corresponding to boundaries where there is a 95%, 90%, 80%, 50%, 20%, 10%, and 5% probability (from outermost to innermost line) for the position of cells in each cluster based on gaussian kernel density estimation. Side panels show histograms for the actual count of events either in the red (top) or green (side) fluorescence channel.

Because each species has a unique fluorescence profile, we determined whether flow cytometry could distinguish them and accurately quantify their absolute abundances in co-culture using four channels or parameters: forward scatter, side scatter, green fluorescence, and red fluorescence. Using these parameters, multicolor flow cytometry was able to distinguish four of the six *Bacteroides* species (Figure 1). We grew anaerobic monocultures of each *Bacteroides* species for 24 h in rich media and processed samples with flow cytometry as described in section Materials and methods. We randomly selected 25,000 events from each monoculture and combined them post-processing into a single data set. In a bivariate plot of red and green fluorescence, each *Bacteroides* species was characterized by a unique cluster of events (or cells) that can be distinguished from other species (Supplementary Figure 1A). However, the green fluorescence signals of *B. ovatus* and *B. uniformis* significantly overlapped with signals of *B. eggerthii* and *B. fragilis*, respectively. Discrimination of six species in a co-culture therefore required further optimization of sample processing and flow cytometry parameters.

To improve mapping of flow cytometry events to individual species, we tested additional sample processing steps and focused our experiments on species that were most divergent in fluorescence. First, because both GFP and mCherry proteins need oxygen to fold (Tsien, 1998), we provided longer maturation times in atmospheric air to increase the intensity of fluorescence signals and separate flow cytometry clusters. Although human gut *Bacteroides* are aerotolerant (Tally et al., 1975), we also processed samples at 4°C to diminish any potential physiological responses to oxygen. In a four-species community, clusters of *B. theta* and *B. vulgatus* cells indeed exhibited higher GFP signal intensities with overnight maturation (Figure 1) than with 4–6 h of maturation (Supplementary Figure 1A). Second, to distinguish cells from debris in suspensions, we stained all cultures with cell-permeant SYTO 40 blue fluorescent nucleic acid stain (Thermo Fisher Scientific). After sample acquisition, data are filtered by a pre-determined threshold in the blue channel that separates stained events from non-stained events. Our final gating strategy and instrument configuration are described in Materials and methods in the “Flow cytometry of bacterial culture” section. Finally, we narrowed our model system by excluding species with intermediate expression of GFP because their fluorescence overlapped with the fluorescence of species that both did not express GFP or highly expressed GFP. Our final model community therefore comprised four *Bacteroides* species: *B. vulgatus* expressed both GFP and mCherry proteins; *B. thetaiotaomicron* expressed a GFP protein; *B. fragilis* expressed an mCherry protein; and a wildtype *B. ovatus* that did not express either fluorescence protein, but instead auto-fluoresced in both channels.

### Machine learning accurately predicts species identity

In addition to our experimental optimization, we further enhanced the accuracy of our model system using machine learning. Manual gating is often the primary strategy for quantifying sub-populations of events in a flow cytometry sample, but this approach is laborious, prone to human biases, difficult to reproduce, and often imprecise (Aghaeepour et al., 2013). We observed in monocultures that the intensity of GFP and mCherry signals changed significantly under different experimental conditions (Supplementary Figures 1B,C), including the type of the carbon source in the culture media. Such variation also made gating at pre-determined thresholds unreliable. To overcome the challenges inherent to manual gating, a variety of methods have been developed for automating analysis of flow cytometry samples using reputed statistical and machine learning approaches (Rubbens and Props, 2021). For example, the multivariate clustering approach of Gaussian Mixture Models has been used for cell population identification and cytometric fingerprinting (Ludwig et al., 2019; Rubbens et al., 2021). In addition, machine learning classifiers such as Random Forests have been used to identify known taxonomic identity at the cell level (Rajwa et al., 2008; Rubbens et al., 2017; Özel Duygan et al., 2020). We therefore developed an *ad hoc* tool in which Gaussian Mixture Models identify and quantify overlapping clusters of cells in a sample while Random Forest classifiers determine the taxonomic identity of each cluster based on *a priori* knowledge.

**Figure 2:**
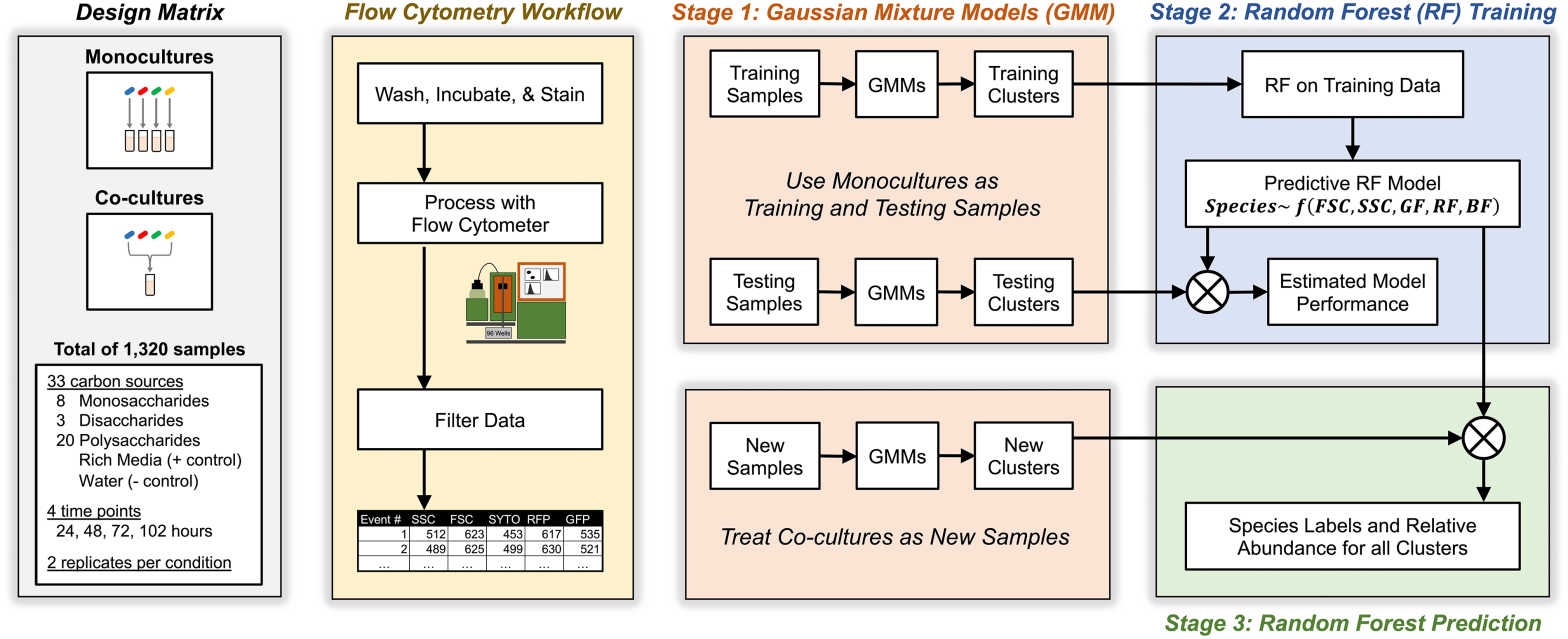
Schematic for experimental, flow cytometric, and machine learning workflows. Study design included monocultures and co-cultures of four *Bacteroides* species on minimal media supplemented with one of 31 carbon substates. At different time points, cultures were sampled, washed, stained, then analyzed with flow cytometry. Flow events were filtered based on pre-determined criteria then analyzed with a three-stage machine learning algorithm that maps flow cytometry clusters in co-cultures to individual *Bacteroides* species.

Our approach relied on a three-stage algorithm (see Figure 2 and Materials and methods for details). In the first stage, we grew each *Bacteroides* species as a monoculture in a variety of different culture conditions for a total of 1,056 unique samples. Then, we analyzed these known monocultures with multicolor flow cytometry which described each event with five cytometric features: forward scatter, side scatter, red fluorescence, green fluorescence, and blue fluorescence. Each cytometric sample was then analyzed with a multivariate Gaussian mixture model which inferred clusters of events in each sample (see Materials and methods for inference of the number of clusters) and described these clusters by their mean values in each of the five cytometric features. In the second stage, these clusters (whose taxonomic identity is known) were then fed to a machine learning classifier using Random Forests that learns to associate each *Bacteroides* species with the observed distribution of its clusters as described by the five cytometric features. In the third stage, we similarly identified clusters in co-culture samples (of two or more *Bacteroides* species) with Gaussian Mixture Models and then predicted the species identity of these clusters with the Random Forest classifier that had been trained in the second stage. Model predictions were then used to compute the absolute abundance of each of the four *Bacteroides* species in all co-cultures.

Because we measured the growth of species on each substrate with two experimental replicates, we were able to train our model on one of those replicates and test the model’s performance on the other. We estimated from these tests that our approach had a small error rate of 5.9% indicating very high model performance (see Materials and methods for details). The most important features for the model were the intensities of red and green fluorescence for each cluster (Supplementary Figure 2A). Prediction error ranged between 6.2% and 6.9% for *B. ovatus*, *B. theta*, and *B. vulgatus*, but was only 3.4% for *B. fragilis* (Supplementary Figure 2B). Clusters that were incorrectly predicted had lower cell counts, lower cluster weights, and lower prediction probability than correctly predicted clusters (Supplementary Figures 2C–E). We confirmed that model performance increased when data were filtered to remove samples with low cell counts (due to poor or no growth), or clusters with low weights (due to debris or alternative physiological states; Supplementary Figures 2F,G). These patterns suggested that errors were biased toward clusters that occur at low abundances in low biomass samples. Nonetheless, the final machine learning model was trained on all clusters regardless of their cell counts or weights.

In summary, we developed a multicolor flow cytometric approach with a machine learning classifier that can accurately track species abundances in a small, synthetic community. By using a flow cytometer with an automated plate handler, we can rapidly screen our synthetic community for interactions between different species under a vast number of conditions.

### Growth on limited nutrients to detect cross feeding interactions

Having constructed an experimental and computational platform for quantifying abundances in a synthetic community of *Bacteroides* species, we next profiled how our community grew in a nutrient-limited environment. When a microbial community encounters a single carbon substrate, its members may either compete for that substrate, which we refer to as resource competition (Ghoul and Mitri, 2016), or share the substrate, which we refer to as cross feeding (Smith et al., 2019). We thus screened for competition and cross feeding between *Bacteroides* by growing each species individually in monocultures and all species collectively in co-cultures in media supplemented with only one of 31 carbon substrates for a total of 1,320 samples (Figure 2; Supplementary Table 2). We also tested growth of all monocultures and co-cultures on rich media as a positive control and minimal media as a negative control. At 48 h, cultures were serially passaged in fresh media and monitored for an additional 48 h. All experimental conditions were sampled daily for flow cytometry analysis. For each substrate, we defined the “maximum monoculture productivity” as the highest concentration (cells per ml) reached by any species in monoculture at any of the sampled time points and defined the “maximum co-culture productivity” as the highest concentration (cells per ml) reached collectively by all species in co-cultures at any of the sampled time points. By tracking microbial growth on diverse substrates, including monosaccharides, disaccharides, and polysaccharides, we could further test whether cross feeding behavior was affected by the identity and complexity of the carbon source.

### Competition for substrates limits community productivity but increases diversity

**Figure 3:**
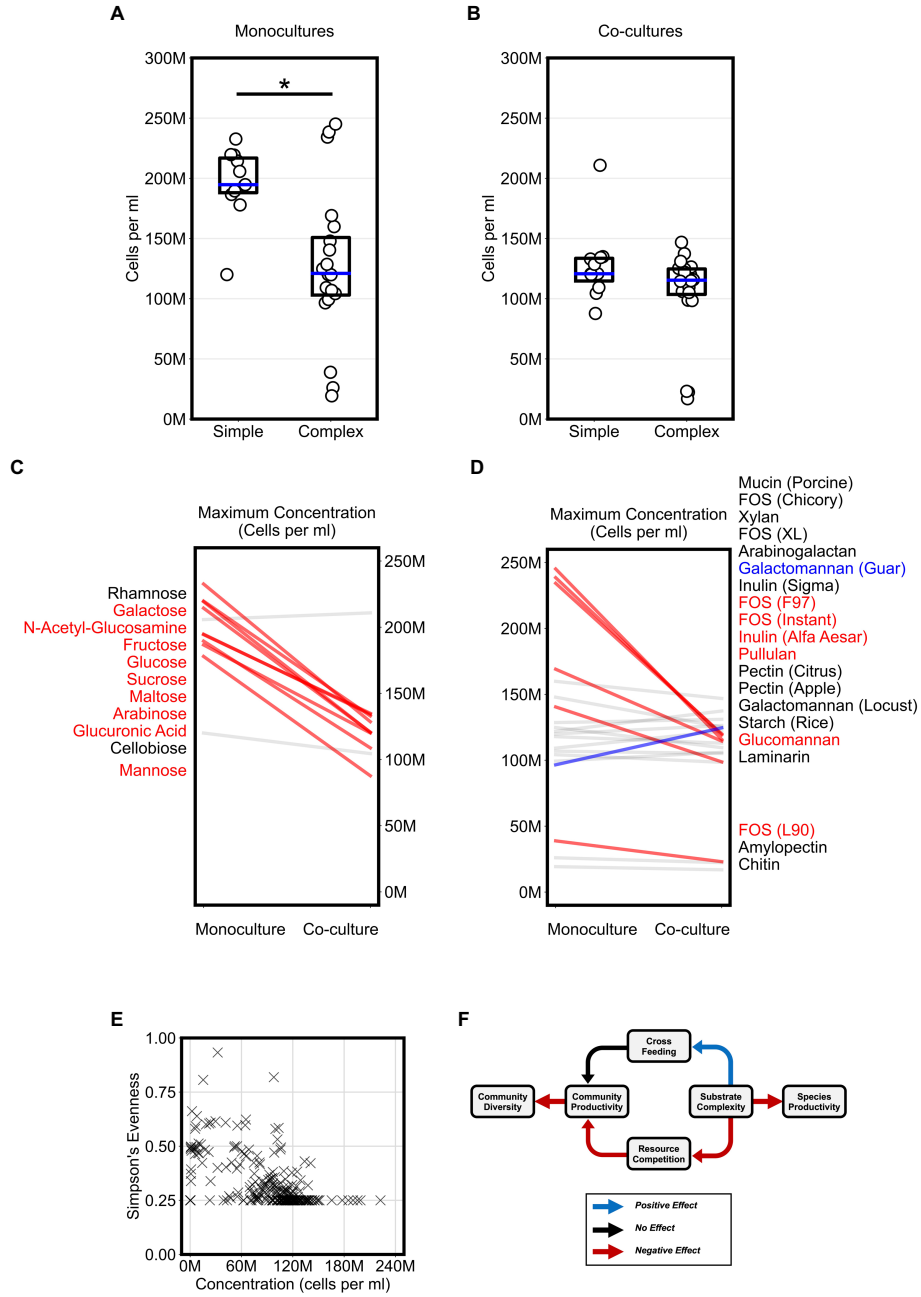
Competition for substrates lowers community productivity but increases diversity. **(A,B)** Plots display the “maximum monoculture productivity” and “maximum co-culture productivity” on each of the tested simple and complex substrates. Boxplots show the range between the first and third quartiles and blue lines indicate medians. Each data point is an average of two experimental replicates. Asterisks indicate an adjusted *p*-value < 0.05 for Bonferroni-corrected nonparametric Mann–Whitney *U*-tests. **(C,D)** Maximum productivity of monocultures and co-cultures separated by simple and complex sugars, respectively. Absolute abundances (cells per ml) in co-cultures that are at least 20% higher or lower than their corresponding abundances in monocultures are highlighted with thicker lines and colored either blue or red, respectively. Substrate annotations are ordered by abundance values in co-cultures. **(E)** Scatter plot of community evenness against absolute abundance for all co-culture samples in this study. **(F)** Schematic for a working model of the impact of substrate complexity on microbial interactions, productivity, and diversity. In all plots, “M” indicates values in the millions.

To evaluate the impact of substrate complexity on microbial productivity, we broadly compared the productivity of monocultures and co-cultures on all 31 substrates. First, we assessed if substrate complexity influenced monoculture productivity. Maximum monoculture productivity was significantly higher on simple sugars than complex sugars (Figure 3A, Bonferroni-corrected Mann–Whitney *U*-test, *p* < 0.01). To test whether this was a common phenotype among a broader set of *Bacteroides* species, we reanalyzed a publicly available dataset that reported the growth of 354 Bacteroidetes isolates, including 328 *Bacteroides* isolates, from the human and animal guts on 45 carbon substrates using spectrophotometry (Pudlo et al., 2022). Again, we found that most isolates reached a higher average growth yield on simple sugars than on complex sugars (Supplementary Figures 3A,B). The higher growth on simple sugars suggested that *Bacteroides* species convert simple sugars to biomass more efficiently than complex sugars in short batch culture growth. In contrast, we did not observe a significant difference in maximum productivity between co-cultures grown on simple sugars vs. those grown on complex sugars (Figure 3B). In fact, the maximum co-culture productivity on simple sugars was lower than the maximum monoculture productivity achieved on simple sugars (Figures 3A,B, Bonferroni-corrected Mann–Whitney *U*-test, *p* < 0.01). The lower productivity for co-cultures on simple sugars suggested that species may compete for simple sugars in a manner that harmed their overall productivity.

Next, we wanted to classify the co-cultures of all four *Bacteroides* species on each substrate based on whether growth on a substrate promoted competition or cross feeding that significantly altered productivity. For each substrate, we expected that competition could result in co-cultures that yield lower abundances than what can be achieved individually by any of the members of the community on their own. In contrast, we expected that cross feeding could result in co-cultures that yield higher abundances than what can be achieved by any of the members of the community on their own. In our co-cultures, productivity significantly decreased by at least 20 percent when compared to monocultures grown on most simple sugars (82% of simple sugars) and did not increase for any of the simple sugars (Figure 3C). However, co-cultures grown on complex sugars displayed fewer decreases in productivity (32% of complex sugars) when compared to monocultures and an increase in productivity on only one of the complex sugars (Figure 3D). Hence, our data suggested that co-culturing on simple sugars showed stronger signs of competition than co-culturing on complex sugars.

Because co-cultures grown on complex sugars displayed fewer signs of resource competition than co-cultures on simple sugars, we hypothesized that substrate complexity may accordingly promote microbial diversity in our model system. Using our flow cytometric approach with a machine learning classifier (see Materials and methods), we first inferred the absolute abundance of each species in co-cultures then estimated community diversity using Simpson’s Evenness index. However, our analysis did not reveal an association between microbial diversity and substrate complexity as we expected. Instead, we found that microbial diversity was negatively associated with community productivity (Logistic Regression, *p* < 0.001), suggesting that less productive communities were more diverse (Figure 3E). In summary, our analysis suggested a working model (Figure 3F) in which simpler substrates promote a more competitive environment during co-culturing which consequently yields less productive communities but allows for higher microbial diversity whereas complex substrates curtail competition and therefore indirectly promote productivity.

### *Bacteroides* species show broad consumption of carbon substrates and co-cultures were dominated by *Bacteroides vulgatus*

**Figure 4:**
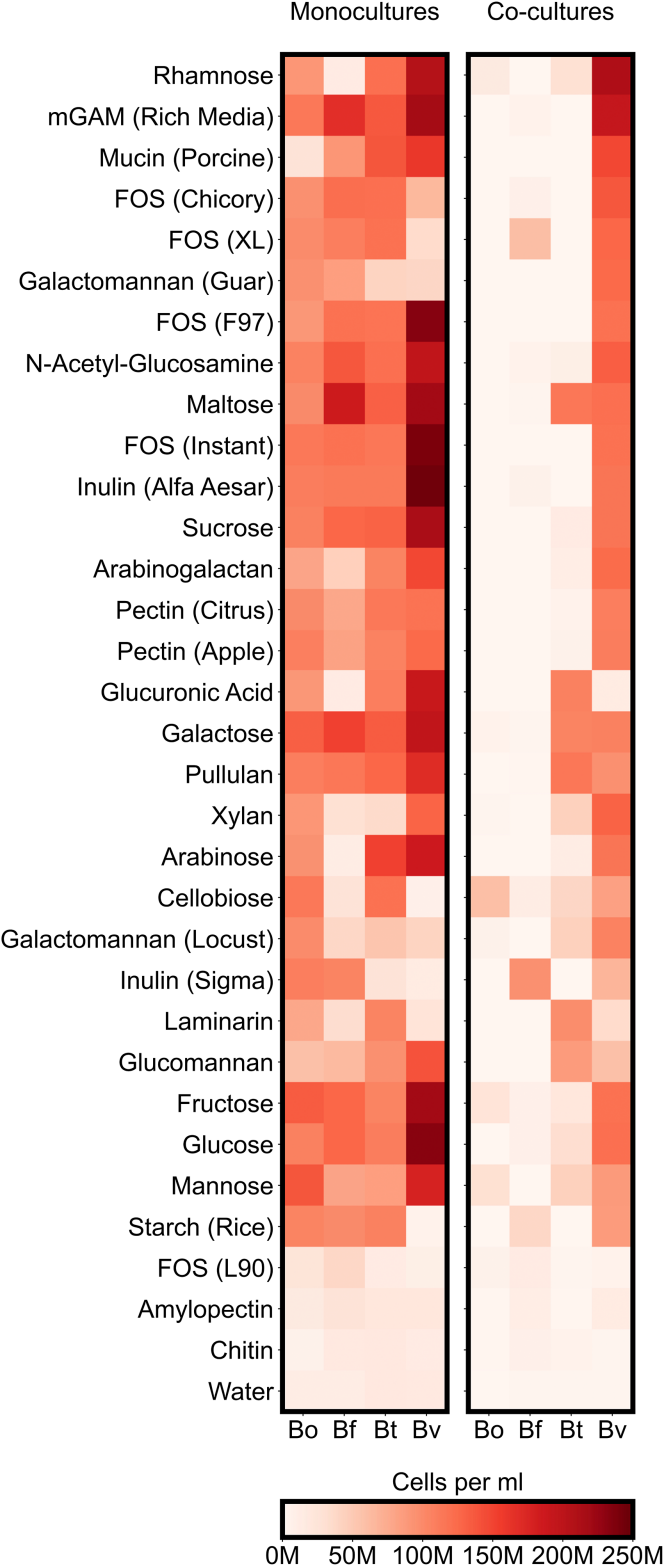
Maximum species concentrations on each tested substrate. For each species, heatmaps display the highest abundances (cells per ml) observed at any of the sampled timepoints either in monocultures or co-cultures. “M” indicates values in the millions.

To better understand how co-culturing various *Bacteroides* species affects productivity and microbial diversity, we next analyzed the growth of individual *Bacteroides* species on the variety of carbon sources in our assay. Our analysis thus far suggested that different *Bacteroides* species are more likely to compete with one other when co-cultured on simple sugars than on complex sugars. These results led us to hypothesize that whereas each simple sugar can be consumed by multiple *Bacteroides* species, complex sugars may only be consumed by only one or two species. We therefore analyzed our flow cytometric data to quantify the growth of each *Bacteroides* species on each substrate. We classified growth on a carbon source as positive if the maximum concentration of a species grown on a particular substrate was at least twice as high as its maximum concentration in the negative control (minimal media supplemented only with water). By this measure, all of the *Bacteroides* species were able to utilize a broad array of simple sugars: 7 substrates (64% of simple sugars) were consumed by all four species and the remaining 3 substrates (36% of simple sugars) were consumed by three species each. Similarly, *Bacteroides* were able to utilize most polysaccharides: 13 substrates (65% of complex sugars) were consumed by all four species, and 3 substrates (15% of complex sugars) were consumed by three species each (Figure 4; Supplementary Figure 4). To verify these growth profiles, we compared our monocultures to carbon substrate utilization of *Bacteroides* species using publicly-available data from plate reader assays (Pudlo et al., 2022). Our results generally agreed with optical density-based assays by Pudlo et al., but our flow cytometry-based data also detected unexpected growth of certain species on several polysaccharides including arabinogalactan, galactomannan, glucomannan, laminarin, pectin, and xylan (Supplementary Figures 3C,D). Because *Bacteroides* species were able to broadly consume both simple and complex sugars, the reduced productivity of co-cultures on simple substrates cannot be explained by the number of species that could utilize each sugar.

Though *Bacteroides* were able to broadly grow on both simple and complex sugars, we next hypothesized that *Bacteroides* were more competitive on simple sugars than on complex substrates. *Bacteroides vulgatus* had significantly higher growth on the simple sugars than the other three species (Supplementary Figures 5A,B, Bonferroni-corrected Mann–Whitney *U*-test, *p* < 0.05) but we found no significant differences in productivity of the four species when comparing growth only on complex sugars. Further, *B. theta* and *B. vulgatus* showed higher average growth on simple sugars than on complex sugars (Bonferroni-corrected Mann–Whitney *U*-test, *p* < 0.05). Therefore, our results suggested that individual *Bacteroides* species are more competitive on simple sugars than complex substrates and that *B. vulgatus* is significantly more competitive on simple sugars relative to other species.

**Figure 5:**
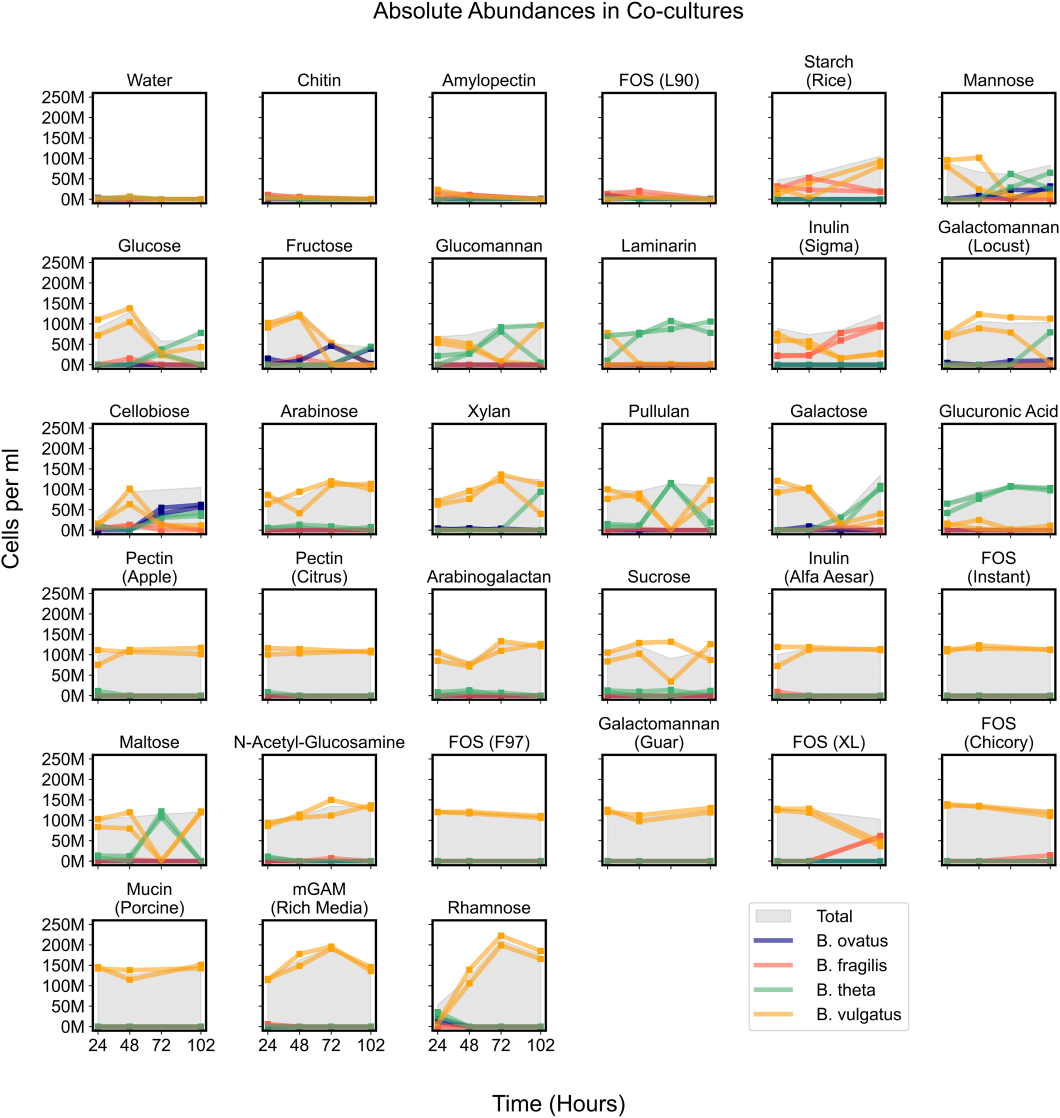
Absolute abundances of *Bacteroides* species in co-cultures on minimal media supplemented with single carbon substrates. Growth curves display absolute abundance of each species at each time point in 34 tested media. Plots include co-cultures on minimal media without a carbon source (Water) as a negative control and co-cultures on rich media (mGAM) as a positive control. “M” indicates values in the millions. Parentheses in titles indicate either the vendor source (e.g., Sigma or Alfa Aesar), organic source (e.g., starch from rice), or proprietary name (e.g., fructooligo saccharides L90). “Total” corresponds to the cumulative concentration (cells per ml) of all species in each sample. Square markers indicate actual measurements which are connected by lines and each line corresponds to a unique experimental replicate. Bo, *Bacteroides ovatus*; Bf, *Bacteroides fragilis*; Bt, *Bacteroides theta*; Bv, *Bacteroides vulgatus*.

Because the growth yields of *Bacteroides* species varied on the same sugars, we also asked if the community productivity in co-cultures could be predicted by the productivity of individual species. We therefore tested for correlations between the maximum abundances of each species in co-cultures with the maximum total abundances of co-cultures and found that *B. vulgatus* abundance is strongly correlated with the total abundances in co-cultures (Bonferroni-corrected spearman’s ρ = 0.92, *p* < 0.001; Supplementary Figure 5C). Indeed, *B. vulgatus* dominated most co-cultures (Figure 5). Because *B. vulgatus* dominated many cultures regardless of substrate complexity, we hypothesized that *B. vulgatus* may be the main driver of resource competition. We therefore tested for correlation between the maximum productivity of *B. vulgatus* in monocultures against the relative change in productivity, defined as ratio of maximum co-culture productivity to maximum monoculture productivity, and found that *B. vulgatus* negatively correlated with relative change in productivity (Bonferroni-corrected Spearman’s ρ = 0.55, *p* < 0.01; Supplementary Figure 5D) such that co-cultures reached lower growth yields on substrates that *B. vulgatus* consumes well and reached higher growth yields on substrates that *B. vulgatus* consumes poorly. Thus, resource competition in our model system for simple sugars may have been most strongly influenced by the fitness of *B. vulgatus*.

While *B. vulgatus* often dominated co-cultures and was the primary contributor to maximum community productivity, we wondered whether other species could coexist with *B. vulgatus* at time points before, during, or after *B. vulgatus* dominance. We observed that communities growing on simple sugars such as cellobiose, fructose, galactose, glucose, and mannose showed initial dominance of *B. vulgatus* followed by coexistence of various species (Figure 5). In addition, *B. vulgatus* initially dominated communities growing on glucomannan and inulin, procured from Sigma, but these cultures were later dominated by *B. theta* and *B. fragilis*, respectively, at later time points (Figure 5; Supplementary Figure 6). The growth of *B. vulgatus* on inulin and cellobiose was striking because *B. vulgatus* was unable to grow as a monoculture on either of those substrates, which indicated potential cross feeding that benefited *B. vulgatus* in co-cultures. In summary, co-cultures were sometimes dynamic such that species composition varied over time, but their productivities were mostly influenced by *B. vulgatus.*

### *Bacteroides vulgatus* solely benefits from cross feeding on complex sugars

**Figure 6:**
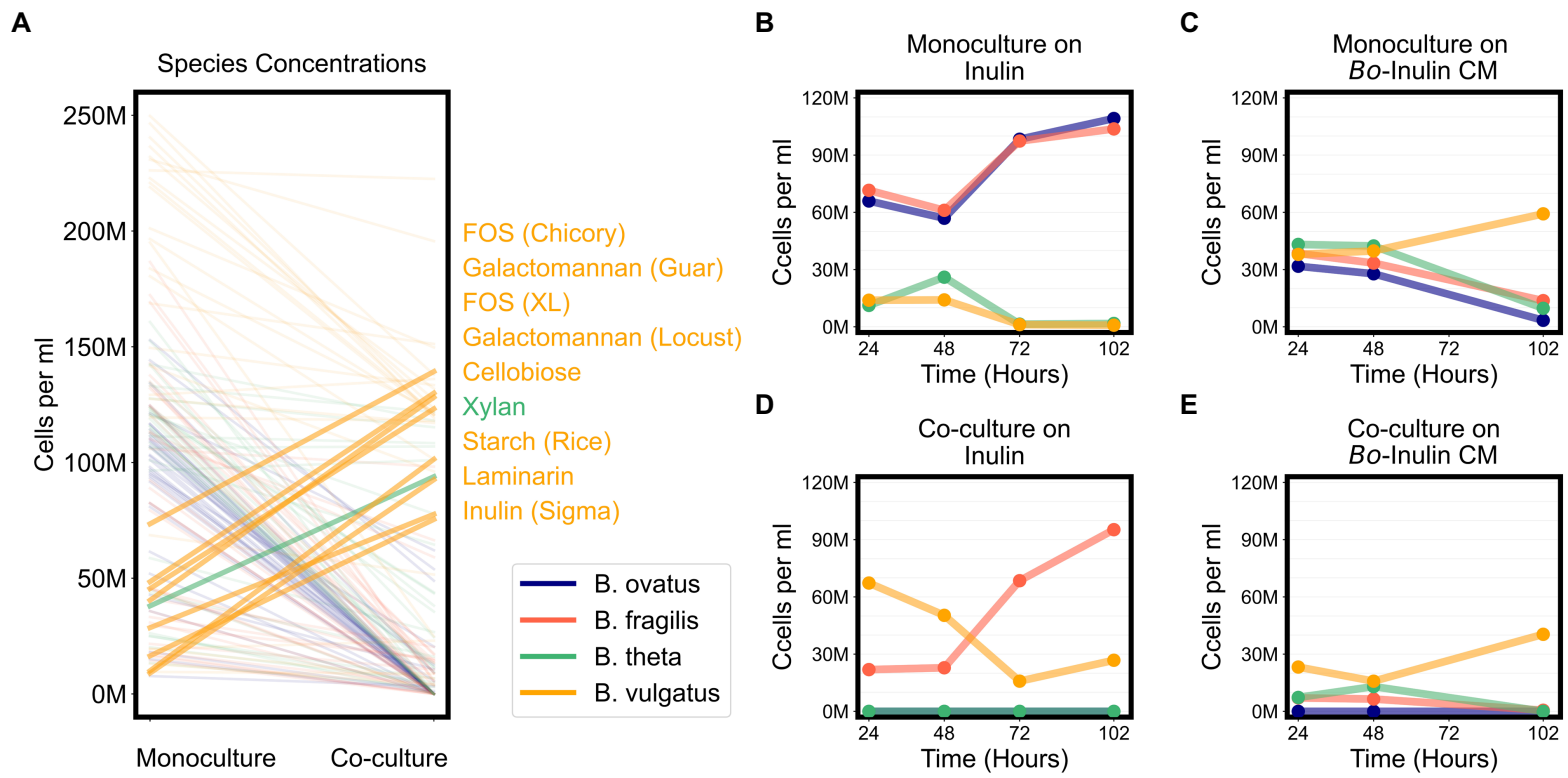
*Bacteroides vulgatus* is the primary beneficiary of cross feeding on complex sugars. **(A)** Lines plot the differences in the maximum abundances (cells per ml) of each of the *Bacteroides* species on each substrate in monocultures vs. co-cultures. Species abundances in co-cultures that are at least 20% higher than their corresponding abundances in monocultures are highlighted with thicker lines and annotated on the right side of the plot. Substrate annotations are ordered by abundance values in co-cultures. **(B,D)** Abundances in monocultures and co-cultures on minimal media supplemented with inulin. **(C,E)** Abundances in monocultures and co-cultures on minimal media supplemented with conditioned media (CM) from the growth of *Bacteroides ovatus* on inulin. In all plots, “M” indicates values in the millions.

Due to signs of cross feeding by *B. vulgatus* in our co-cultures, we tested for additional conditions under which any species reached a higher biomass in co-cultures than in monocultures, even if the overall productivity of co-cultures was not higher than productivity of monocultures. We found that *B. vulgatus* grew to higher biomass in co-cultures on eight substrates; *B. theta* reached slightly higher biomass on galactomannan and xylan; and neither *B. ovatus* nor *B. fragilis* benefited from co-culturing (Figure 6A; Supplementary Figure 5E). Most of the substrates that promoted higher growth of *B. vulgatus* or *B. theta* are polysaccharides that are typically digested by extracellular enzymes released by other species in the community (Figure 6A). For example, *B. ovatus* secretes extracellular enzymes known to break down inulin which then allows other species, including *B. vulgatus,* to grow on the inulin breakdown byproducts even though *B. vulgatus* lack the requisite digestive machinery themselves (Rakoff-Nahoum et al., 2016). Indeed, in our assays, *B. vulgatus* was unable to grow on inulin procured from Sigma-Aldrich in monocultures but grew substantially in co-cultures particularly at earlier time points (Figures 6B,C). We then verified that *B. vulgatus* is able to grow on the degraded inulin found in the filter-sterilized supernatant of a *B. ovatus* monoculture grown on inulin (Figures 6D,E). Notably, we did not see a similar pattern with a different formulation of inulin procured from Alfa Aesar. *Bacteroides vulgatus* was able to grow on this formulation of inulin in both monocultures and co-cultures (Figure 4). Our results indicate that *B. vulgatus* is the primary species that benefits from co-culturing likely due to substrate cross feeding where one species breaks down a complex sugar but shares the byproducts with neighboring species.

## Discussion

Here, we developed a multicolor flow cytometric approach for tracking the abundance of multiple *Bacteroides* species in a high throughput fashion capable of testing a variety of experimental conditions. We detected competitive and cross feeding interactions between these species and observed the impact of resource competition and substrate complexity on the productivity and diversity of their communities. Using a model system of four *Bacteroides* species with fluorescent reporters, we rapidly quantified the abundance of each species in co-cultures using multicolor flow cytometry with a machine learning classifier. Results from monocultures indicated that *Bacteroides* species were individually more productive on simple sugars than complex polysaccharides. Yet, productivity of *Bacteroides* co-cultures on simple sugars was significantly curtailed, possibly due to heightened competitive abilities of the individual species on simple sugars. In contrast, co-culturing on complex sugars showed signs of both competitive and cross feeding interactions and community productivity was only impacted for select complex substrates. Further, *B. vulgatus* fitness was associated with heightened competition such that communities were less productive on substrates that *B. vulgatus* consumed robustly. However, lower community productivity in these instances was associated with coexistence of multiple *Bacteroides* species and, therefore, higher community diversity. Coexistence was also promoted by cross feeding interactions that primarily benefited *B. vulgatus,* which passively consumed nutrients shared by other species that actively digested complex polysaccharides.

Although resource competition is ubiquitous, natural microbial communities show incredible diversity in nutrient-limited conditions. A single monosaccharide can support microbiotas that are highly diverse and taxonomically rich (Flynn et al., 2017; Goldford et al., 2018; Dal Bello et al., 2021). Positive interactions between these microbes are typically mediated through the sharing or exchange of nutrients (Coyte and Rakoff-Nahoum, 2019; Smith et al., 2019). Microbes including *Bacteroides* can thus coexist on limited nutrients through cross feeding, syntrophy, and cooperation (Rakoff-Nahoum et al., 2014, 2016; Ruaud et al., 2020; Pacheco et al., 2021). Substrate complexity can further promote coexistence through niche partitioning by allowing multiple species to actively consume the same polysaccharide (Enke et al., 2019; Brochet et al., 2021). Our results in a small, synthetic community confirmed that a rich cross feeding network exists between *Bacteroides* on single carbon sources. Furthermore, tradeoffs may be embedded within this network. While coexistence on simple sugars is possible, our data suggest overall community productivity can be limited by competition. By contrast, cross feeding on complex sugars allows for coexistence without negatively impacting community productivity.

In our *Bacteroides* model system, the single species *B. vulgatus* was a common driver of both resource competition and cross feeding. By contrast, previous metabolic profiling suggested that *B. ovatus* tends to be a versatile polysaccharide degrader while other *Bacteroides* species such as *B. fragilis*, *B. thetaiotaomicron*, and *B. vulgatus* each degrade a more limited set of polysaccharides (Pudlo et al., 2022). Still, our findings are consistent with reports that multiple *Bacteroides* species, including *B. vulgatus,* can facilitate as well as benefit from cross feeding (Rakoff-Nahoum et al., 2014). Our tests suggested that, when cultured with other *Bacteroides* species, *B. vulgatus* was able to grow on substrates including fructooligosaccharides, galactomannan, inulin, and starch, although it poorly grew on those substrates in monocultures. We confirmed that *B. vulgatus* grew on inulin because other species released extracellular enzymes that degrade inulin into components that *B. vulgatus* then consumed. In several instances, *B. vulgatus* dominated co-cultures on substrates that it cannot individually consume even when other species were limited to low absolute abundances. Although these other species were low in abundance, it is possible that they released functional enzymes that supported *B. vulgatus* growth. *Bacteroides* previously demonstrated an inverse relationship between growth rate and enzyme activity such that rates of polysaccharide degradation were higher at slower growth (Macfarlane et al., 1990). Still, our results indicate that the ecological patterns observed in short batch cultures present a snapshot of a dynamic interplay between species when grown in community. For example, we saw that *B. vulgatus* initially dominated co-cultures on inulin but *B. fragilis*, which can directly consume inulin, bloomed over time, and eventually dominated the co-culture. While nutrient sharing by a species may initially constitute a fitness cost, the public goods producer, such as *B. fragilis*, may have the competitive advantage in the long term over cheaters or cross-feeders. Our multicolor flow cytometry approach can be applied to future experiments to explore why, and over what time-scales, *B. vulgatus* promotes resource competition and dominates mixed *Bacteroides* communities.

Our data indicate that flow cytometry-based growth assays for measuring bacterial abundance can complement traditional methods for measuring microbial growth. Our results broadly agree with published reports of *Bacteroides* substrate utilization (Desai et al., 2016; Pudlo et al., 2022) but also suggest that that the ability of *Bacteroides* to grow on certain polysaccharides may still be underestimated in the field. Differences in substrate chemistry, bacterial growth protocol, and detection method may explain why our data slightly deviate from previously published findings. For example, resource utilization by *Bacteroides* has often been tested by cells harvested in stationary phase, diluted, pre-cultured on minimal media supplemented with glucose, and then inoculated into minimal media with test substrates (Desai et al., 2016; Pudlo et al., 2022). By contrast, cross feeding by *Bacteroides* was heightened by cells harvested during their exponential growth (Rakoff-Nahoum et al., 2014), and, in our preliminary studies, all four species in our model showed more rapid, robust, and similar growth yield on sucrose in contrast to simple sugars such as fructose and glucose. Therefore, we tested resource utilization with cells harvested in mid-logarithmic phase that had been pre-cultured on minimal media supplemented with sucrose. The choice of carbon source in the pre-culture may be significant because *Bacteroides* respond to simple sugars such as glucose by silencing a regulator involved in polysaccharide utilization and colonization (Rakoff-Nahoum et al., 2014). However, the most significant difference between our study and previous reports of *Bacteroides* growth may be due to strengths of our detection method. Flow cytometry may be a more sensitive method of quantifying growth because it measures growth at the level of individual cells whereas spectrophotometric methods only estimate bulk growth based on optical density (Adan et al., 2017). Further, flow cytometry can easily distinguish between live and dead cells if samples are stained with a live/dead stain at the time of collection (Buysschaert et al., 2016; Duquenoy et al., 2020). The accuracy of spectrophotometry is also sometimes limited to a high detection limit and a small optical range which is further constrained by the background turbidity of the media (McGoverin et al., 2021). Fibers with low solubility such as laminarin and xylan (Guo et al., 2017) can alter the opacity of media and thus complicate the accurate detection of microbial growth (Wang et al., 2010). Indeed, the limitations of spectrophotometry for detecting growth on diverse substrates motivated our flow cytometric approach for quantifying *Bacteroides* growth.

Our technique should be particularly useful in bottom-up ecological modeling with aero-tolerant bacterial species in synthetic communities. Aerotolerant species are ideal for these experiments because exposure to atmospheric levels of oxygen is not toxic, and incubation for several hours at atmospheric air is sufficient to allow fluorophore folding anoxically. Also, as an added advantage, our approach requires minimal sample processing prior to flow cytometry. Unlike molecular or sequencing methods, this method does not require extracting, amplifying, and purifying DNA, which allowed us to rapidly analyze a total of 1,620 samples within 24 h of the completion of our growth assays While an experiment of this scale could have been analyzed with sequencing, our flow cytometric approach is significantly more efficient (all samples were processed with 24 h of collection) and less expensive (costs are estimated at $3.30 per sample if using a university flow cytometry facility) utilizing fewer reagents and less human labor. Further, our technique inherently estimated the absolute abundance of species without any additional processing such as DNA spike-ins (Stämmler et al., 2016) or qPCR analysis. In future experiments, we plan to further evaluate and improve the accuracy of our approach using mock community experiments, benchmarking against alternative robust species counting approaches (e.g., fluorescence imaging). Our flow cytometry approach can also be expanded to track more than four species. Recent dramatic increases in the number of fluorochromes, the number of fluorescence detectors, and embedded image-based analysis have facilitated flow cytometry experiments with dozens of parameters (McKinnon, 2018; Ugawa et al., 2021; Schraivogel et al., 2022). Our approach can thus be easily scaled to include additional species, evaluate more complex environments, and assay response of communities to a variety of disturbances. Altogether, these advancements will facilitate bottom-up ecological modeling that can test important ecological principles of how microbial communities assemble and interact under a variety of conditions in an efficient, high-throughput manner.

## Materials and methods

### Growth media

*Bacteroides* species were grown either on Blood Agar Plates (Hardy Diagnostics), a modified Gifu Anaerobic Medium (mGAM), or a defined minimal medium. mGAM is a Gifu Anaerobic Medium (HiMedia Laboratories) with 1% w/v hemin and 5% w/v menadione (Rettedal et al., 2014). These additional micronutrients of iron and vitamin K_3_ improve the cultivation of several *Bacteroides* species (Gibbons and Macdonald, 1960). The minimal medium was made as previously described (Villa et al., 2020). One liter of minimal medium contained 100 mM KH_2_PO_4_ (pH 7.2), 15 mM NaCl, 8.5 mM (NH_4_)_2_SO_4_, 4 μΜ L-cysteine, 1.9 μΜ hematin, 200 μΜ MgCl_2_, 1.4 μΜ FeSO_4_•7H_2_O, 50 μΜ CaC_2_, 1 μg mL^−1^ vitamin K_3_. Minimal medium solution was supplemented with 5% v/v of vitamins (ATCC MD-VS), 5% v/v Trace Minerals (ATCC MD-TMS), 50% v/v of 1:1 Purines:Pyrimidines solutions (Sigma), and 100% of v/v MEM Amino Acids solutions without L-glutamine (Sigma AA-5550), brought to final pH of 7.2, filter sterilized (0.4 μm pore size), and stored in the dark at room temperature.

### Bacterial strains and preparation

List of strains and expected fluorescence proteins are reported in Supplementary Table 1. We acquired strains from the Sonnenburg lab (Stanford University), who engineered reference *Bacteroides* strains (ATCC) by inserting chromosomally integrated fluorescence reporters (Whitaker et al., 2017). Frozen glycerol stocks of *Bacteroides* were thawed for ~30–60 min, streaked on Blood Agar Plates, then grown overnight for ~48 h at 37°C anaerobically (85% N_2_, 10% CO_2_, 5% H_2_) in a vinyl anaerobic chamber (Coy Laboratory Products). Strains were subcultured and passaged twice on agar using the same conditions before any of the experiments included in this manuscript.

### Bacterial growth for carbohydrate utilization assays

For bacterial growth, all consumables and reagents were pre-reduced in an anaerobic chamber before use for a minimum of 2 h. Solutions for each carbon substrate in Supplementary Table 1 were prepared as 1% w/v stocks in purified water (Millipore Milli-Q). Deep 96-wells were arrayed with 300 μl of 1% w/v carbon substrate stocks, transferred to the anaerobic chamber, and allowed to equilibrate for at least 2 h. Bacterial cell suspensions were prepared by swabbing colonies from agar plates into 2 ml of liquid mGAM. Cultures were incubated for ~6–8 h at 37°C in 14 ml round-bottom tubes inside an anaerobic chamber. Cultures were then washed once in minimal medium by centrifugation at 12,000 ×g for 2 min (Eppendorf MiniSpin), resuspended in 2 ml minimal medium supplemented with 0.5% w/v sucrose, and anaerobically grown for 24 h at 37°C. Washing, including centrifugation, was performed inside the anaerobic chamber. To estimate bacterial growth, absorbance values for 200 μl samples were measured at 600 nm using a plate reader (CLARIOstar, BMG Lab Tech Inc.). Based on estimates of bacterial growth, bacterial cultures were washed twice in minimal medium and diluted to an optical density of approximately 0.1 in twice-concentrated minimal medium. For monocultures, these cell suspensions that were diluted to an optical density of 0.1 were used to inoculate corresponding wells in the 96-well plate. For co-cultures, a 14 ml of cell suspension was first prepared by mixing 3.5 ml from the cell suspensions of each of the species, resulting in an equal volume mixture of all four *Bacteroides* species, which was subsequently used to inoculate corresponding wells in the 96-well plate. Cell suspensions of 300 μl were then added to appropriate wells in the 96-well plates bringing the final concentration of carbon substrates to 0.5% w/v and starting optical density of cells to 0.05. Volumes of 50 μl of mineral oil was added to each well to prevent evaporation of culture. Plates were incubated at 37°C for 48 h in the anaerobic chamber. Samples were collected at 24 and 48 h by mixing cultures with pipetting up-and-down prior to transferring 200 μl from each well to another 96-well plate for downstream sample processing. At 48 h, cultures were then restarted by diluting cultures into sterile media. In particular, deep 96-well plates were pre-arrayed with 300 μl of twice-concentrated minimal medium mixed with 300 μl of 1% w/v carbon substrate solutions. Volumes of 30 μl of bacterial culture at 48 h were mixed by pipetting up-and-down into the new wells for a final seed ratio of 1:20 and carbon concentration of approximately 0.5% w/v. Samples were collected at 72 and 102 h by mixing cultures with pipetting up-and-down prior to transferring 200 μl from each well to another 96-well plate for downstream sample processing. Each growth condition, i.e., culture of a specific *Bacteroides* species or co-culture on a carbon substrate, was repeated in duplicates. As a negative control, 300 μl of twice-concentrated minimal medium (no cells in suspension) was mixed with equal volume of twice-concentrated 1% w/v carbon substrate solution.

### Flow cytometry of bacterial cultures

Samples of 200 μl from carbon substrate utilization assays were transferred into 1.5 ml microfuge tubes prefilled with 800 μl of phosphate buffer saline (PBS). Tubes were centrifuged at 10,000 ×g for 4 min at 4°C. Supernatant was decanted without disturbing the pellet and discarded. Pellets were resuspended with 1 ml of cold filter-sterilized PBS, vortexed for at least 5 s, and incubated overnight at 4°C, which allows sufficient time for oxygen-dependent folding of both GFP and mCherry proteins (Tsien, 1998). Tubes were then centrifuged at 10,000 ×g for 4 min at 4°C. Supernatant was decanted without disturbing the pellet and discarded. Pellets were resuspended with 1 ml of 200 μl cold filter-sterilized PBS mixed with 3 μM SYTO 40 blue fluorescent nucleic acid stain (Thermo Fisher Scientific), vortexed for at least 5 s, and transferred to 96-well plates. Flow cytometry only recorded events in a 3 μl sample from each well in these plates.

Flow cytometry of bacterial suspensions was performed with a MACSQuant VYB (Militenyi Biotec) equipped with an automated multi-well plate handler. Fluorescence was excited at 488 nm and monitored using B1 (520/50) nm channel for GFP fluorescence, excited at 561 nm and monitored using Y2 (615/20 nm) channel for mCherry fluorescence, and excited at 405 nm and monitored using V1 (450/50 nm) channel for SYTO 40 fluorescence. Parameters of forward (561/10 nm) and side (561/10 nm) scatter were estimated by excitation at 561 nm. For data recording, we set area of the SSC signal as the primary trigger with a threshold of 1 arbitrary unit and area of the FSC signal as the secondary trigger at a threshold of 1 arbitrary unit. To filter out background events that can be attributed to instrumental noise, disintegrated cells, or media particulates, we only retained events with nonzero signals in all channels and a blue fluorescence signal in the V1 channel above a threshold of 450 arbitrary units which was optimized for our acquisition settings. The total count of events for each sample was the number of events that remained after applying these thresholds.

### Inference of species relative abundances from flow cytometry data

Flow cytometry analysis of a bacterial monoculture typically results in a single cluster of events in the two-dimensional space of green and red fluorescence channels. The expected location of this cluster depends on whether the respective bacteria encode GFP or mCherry. While mCherry and GFP proteins are chromosomally integrated in certain bacteria, these proteins may not properly fold or fully mature, or may partially disintegrate at the time of measurement, due to either experimental or physiological conditions (e.g., cellular growth stage). Therefore, flow cytometry analysis may detect more than one cluster for each monoculture. For example, flow cytometry events of *B. vulgatus*, which encodes both GFP and mCherry proteins, can fall into one of four clusters depending on the fluorescence activity of each individual cell as follows: cells that are positive for GFP and mCherry; cells that are GFP positive; cells that are mCherry positive, and cells that are negative for both proteins. Accordingly, monocultures from distinct species can yield flow cytometry clusters that overlap in the two-dimensional space of green and red fluorescence. We could not distinguish such flow cytometry clusters from distinct species by linear gating. To address this, we implemented a three-stage algorithm that (1) groups events into clusters using Gaussian Mixture Models, (2) learns the expected cluster locations for each species based on monocultures, and (3) maps each cluster in a co-culture to a species using the model trained on monocultures.

#### Stage 1: Within-sample clustering

For each experimental sample, we randomly selected 10,000 events and split them into a training and testing set in an 80:20 ratio. We ran a Gaussian Mixture Model (GMM) on the five variables describing each flow cytometry event: side scatter (SSC-H), forward scatter (FSC-H), green fluorescence (GFP-H), red fluorescence (mCherry-H), and blue fluorescence (SYTO-H), where H indicates height of the corresponding signal. To identify the optimal number of clusters for each sample, we trained multiple GMM models on each training set using a different number of clusters for each model. For *B. ovatus*, *B. fragilis*, and *B. theta*, we expected either 1 or 2 clusters; for *B. vulgatus*, we expected between 1 and 4 clusters; and for the four-species co-cultures, we set an upper limit of 10 clusters accordingly. For sterile media controls with at least 100 events, we only expected one cluster. We then evaluated these multiple models by predicting labels of samples in the testing set and computing the Bayesian Information Criterion (BIC) as a proxy for GMM model fit. The optimal number of clusters corresponded to the GMM model that maximized the BIC on the testing set. We then re-trained the GMM with the optimal number of clusters on all sampled events (training and testing sets combined). For each sample, the final output of this stage is a set of predicted clusters, each of which is described by the mean vector and covariance matrix for the five variables used in clustering. In addition, GMMs assigned weights to each cluster which corresponded to the relative contribution of each cluster to the total number of sampled events.

#### Stage 2: Model learning

In this stage, we trained a Random Forests (RF) model that learned the unique characteristics of the set of clusters belonging to each species. Because all experimental conditions were repeated in duplicate, we used one of those replicates for training the RF model and one for testing it. For the training set, each sample had already been mapped to the appropriate set of clusters using GMMs and its label (species identity) was known because each culture was seeded with a single bacterial species. An RF classifier using 1,000 trees was thus trained and then tested on the second replicate.

#### Stage 3: Model prediction

We applied the trained RF classifier on the co-culture samples. In particular, the RF classifier predicted species identity for each cluster in a sample. After assigning all clusters in a co-culture to species, the sum of weights of clusters assigned to each species corresponded to its relative abundance in the co-culture.

### Inference of species absolute abundances from flow cytometry data

Using volumetric cell counting, we injected a fixed volume of 3 ul from each sample into the flow cytometer and then applied our gating strategy to count cells in this fixed volume. Next, we normalized these cell counts by volume to estimate the absolute abundance expressed as cells per ml. We also multiplied these estimated absolute abundances by a correction factor of five since all samples were diluted 5-fold prior to flow cytometry. We also applied this approach to duplicate sterile samples of each media to estimate background events that may be falsely labeled as cells. For monocultures, we corrected the absolute abundances of cells by simply subtracting the estimated absolute abundances of background events in sterile media. For co-cultures, however, background events bias absolute abundances in a species-specific manner because background events can display autofluorescence. Therefore, we first estimated the absolute abundances of species in each sterile media as described in the previous section, then subtracted the absolute abundances of species-specific background events from the absolute abundances of each species in co-cultures.

### Statistical analysis

Univariate statistical tests with the Mann–Whitney *U*, and Spearman’s ρ were performed with “scipy.stats.mannwhitneyu” and “scipy.stats.spearmanr” functions, respectively. *p*-values were adjusted for multiple comparisons with the Bonferroni-Hochberg procedure. For flow cytometry plots, Gaussian kernel density estimation was performed using scipy.stats.gaussian_kde. We performed linear mixed effects model analysis to determine the effect of different variables on community diversity using “R” version 4.0.2. *p*-values were obtained by likelihood ratio tests comparing the full model (Simpson’s Evenness ~ log10(Community Productivity) + Substrate Complexity +1|Timepoint) with reduced models and were performed with the “anova” function in the “lme4” package. Comparison with reduced models determined that substrate complexity does not affect diversity. Correlation between community productivity and diversity were assessed with logistic regression using the “glm” function with a “gaussian” family and “logit” link.

## Data availability statement

The datasets presented in this study can be found in online repositories. The names of the repository/repositories and accession number(s) can be found at: https://doi.org/10.5281/zenodo.6399495 and https://flowrepository.org/id/FR-FCM-Z5XM.

## Author contributions

FM developed methods, collected data, curated data, analyzed data, and wrote the original draft of the manuscript. FM and LD conceptualized the study, designed analyses, interpreted the results, and edited the manuscript. All authors contributed to the article and approved the submitted version.

## Funding

FM acknowledges support from NSF DGE-1545220 and NIH T32DK007664. LD acknowledges support from the Global Probiotics Council, a Searle Scholars Award, the Hartwell Foundation, an Alfred P. Sloan Research Fellowship, the Translational, Research Institute through Cooperative Agreement NNX16AO69A, the Damon Runyon Cancer Research Foundation, the Burroughs Wellcome Fund Investigators in the Pathogenesis of Infectious Disease program, and NIH 1R01DK116187. Computational resources were supported by the grant 2016-IDG-1013 (HARDAC+: Reproducible HPC for Next-Generation Genomics) from the North Carolina Biotechnology Center.

## Conflict of interest

LD previously served on the Strategic Advisory Board and held equity in the company Klaeido Biosciences.

The remaining author declares that the research was conducted in the absence of any commercial or financial relationships that could be construed as a potential conflict of interest.

## Publisher’s note

All claims expressed in this article are solely those of the authors and do not necessarily represent those of their affiliated organizations, or those of the publisher, the editors and the reviewers. Any product that may be evaluated in this article, or claim that may be made by its manufacturer, is not guaranteed or endorsed by the publisher.

## Supplementary material

SUPPLEMENTARY TABLE 1 List of bacterial strains used in this study.

SUPPLEMENTARY TABLE 2 List of carbohydrates or supplements used in growth assays in this study. NA indicates “Not Applicable.”

SUPPLEMENTARY FIGURE 1 Flow cytometry distinguishes multiple fluorescent Bacteroides species. **(A)** Flow cytometry analysis of events (total of 25,000 events per species) from six species pooled together post-processing. Species were grown overnight in rich media then washed and incubated in aerobic conditions for 6 hours prior to analysis with flow cytometry. Cellular events were characterized by height of their signals either in green (GFP-H) or red (mCherry-H) fluorescence channels. The two-dimensional panel shows contour lines corresponding to boundaries where there is a 95%, 90%, 80%, 50%, 20%, 10%, and 5% probability (from outermost to innermost line) for the position of cells in each cluster based on gaussian kernel density estimation. Side panels shows histograms for the actual count of events either in the red (top) or green (side) fluorescence channel. **(B)** Flow cytometry clusters in monoculture samples exhibited variation in their fluorescence intensities. Here, each data point is a cluster centroid which corresponds to the mean intensity of GFP and mCherry for all events in a cluster. Clusters correspond to monocultures sampled at four different time points (24, 48, 72, and 102 hours) and grown on 34 different media conditions used in this study. For each monoculture sample, we only show the primary cluster that corresponds to the cluster with the highest weight (as estimated by Gaussian Mixture Models). **(C)** Two-dimensional flow cytometry histograms for monoculture of *B. vulgatus* grown on minimal medium supplemented with fructose. Here, multiple clusters can correspond to cells from the same monoculture as seen for 24 hours and 48 hours, and centroids of these clusters can shift over time.

SUPPLEMENTARY FIGURE 2 Random Forest model assigns species identity to flow cytometric clusters with high accuracy. **(A)** Mean and standard deviation for the importance of features used by the Random Forest classifier as estimated by a permutation test (“permutation_importance” function in the “inspection” module of “scikit-learn” version 0.24.0). **(B)** Plot shows counts and directions of prediction errors for each species. Arrows indicate direction of errors from the true species label to the incorrect species label. Thickness of each arrow is proportional to the count of corresponding errors which are also written adjacent to the arrows. *Bacteroides fragilis* had the lowest prediction error while *B. vulgatus* had the highest number of errors but it also had the highest number of clusters in the testing data set. Percentages in the titles indicate proportion of species-specific labels that were incorrectly labeled. **(C-E)** Violin plots describe differences between clusters that were correctly and incorrectly labeled in hold-out validation. Clusters that were correctly predicted **(C)** tended to belong to samples with lower concentrations (cells per ml), **(D)** had lower weights (which has a range of 0-1), and **(E)** had lower prediction probability. Differences in the distributions of each parameter were tested using the nonparametric Mann-Whitney U statistic. *** indicates that Bonferroni-corrected P-values were less than 0.001. **(F-G)** For evaluating the performance of the Random Forest models, clusters were excluded from model testing and training based on either **(F)** their cluster weight or **(G)** the total concentration (cells per ml) of their parent sample. Learning curves show how the performance of a random forest classifier changes based on adjusting the thresholds for these two criteria. Right vertical axes of these plots indicate the impact of filtering clusters based on either criterion on the sample size (i.e., total number of clusters included in both testing and training data sets) which is plotted in red.

SUPPLEMENTARY FIGURE 3 Comparison of monoculture assays between flow cytometry-based measurements in this work and spectrophotometry-based measurements by Pudlo et al. **(A)** Scatter plot for the average species species productivity of *Bacteroides* isolates on 11 and 20 polysaccharides based on flow cytometric assay used in this study. Colors indicate species: *B. ovatus* (blue), *B. fragilis* (red), *B. thetaiotaomicron* (green), *B. vulgatus* (gold). **(B)** Scatter plot for the average species productivity of *Bacteroides* isolates on 15 monosaccharides and 30 polysaccharides based on spectrophotometric assays by Pudlo et al. Here, we color data points belonging to the parental type strains for the isolates used in this study. Circles that are above the dashed line correspond to isolates that had higher average growth on monosaccharides than on polysaccharides. Averages were computed only on substrates that supported the growth of each isolate. **(C,D)** Comparison of the results of the growth assays in this study and assays in Pudlo et al. (2022). Pudlo and colleagues displayed growth data in heatmaps after normalizing the optical density values for each species such that the growth on the substrate that provided maximum total growth for each strain was set to 1. To fairly compare growth data in our manuscript to the growth data in Pudlo et al. (2022), we applied a similar normalization scheme. Therefore, **(C)** displays maximum absolute abundances which were normalized for each species to a range of 0 to 1, and **(D)** displays optical density values which were normalized for each species to a range of 0 to 1. In all plots, “M” indicates values in the millions.

SUPPLEMENTARY FIGURE 4 Absolute abundances of *Bacteroides* monocultures on minimal media supplemented with single carbon substrates. Growth curves display the median absolute abundance of each species at each time point in 34 tested media. Grid plot includes co-cultures on minimal media without a carbon source (Water) as a negative control and co-cultures in rich media (mGAM) as a positive control. “M” indicates values in the millions. Parentheses in the title indicate either the vendor source (e.g., Sigma or Alfa Aesar), organic source (e.g., starch from rice), or proprietary name (e.g., fructooligosaccharides L90).

SUPPLEMENTARY FIGURE 5 *Bacteroides vulgatus* dominates co-cultures, impacts community productivity, and benefits from cross feeding. **(A,B)** The maximum abundances for each species split by substrate complexity in monocultures and co-cultures respectively. **(C)** Plot shows the maximum abundance of *B. vulgatus* in co-cultures against the maximum total abundance of the four-member community in co-cultures. **(D)** Plot shows the maximum abundance (cells per ml) of *B. vulgatus* in monocultures against the relative change in productivity, defined as the ratio of “maximum co-culture productivity” to “maximum monoculture productivity,” for each substrate. **(E)**. Plot shows the maximum productivity (cells per ml) of each species in co-cultures vs. monocultures for all substrates. Shaded blue and red regions indicate growths in co-cultures that are at least 20% higher or lower than monocultures respectively. Colors indicate species: *B. ovatus* (blue), *B. fragilis* (red), *B. thetaiotaomicron* (green), and *B. vulgatus* (gold).

SUPPLEMENTARY FIGURE 6 Absolute abundances of *Bacteroides* species in co-cultures on minimal media supplemented with single carbon substrates. Heatmaps display the average abundance of each species in co-cultures. For each substrate, the left heatmap displays the relative abundances of each species at each time point, and the right heatmap displays the absolute abundance of the whole community at each time point. Absolute abundances correspond to the total cell concentration (CC) in cells per ml and “M” indicates values in the millions. Heatmap cells highlighted with “X” correspond to missing samples.
