# Supplementary figures and images for: Tracking defined microbial communities by multicolor flow cytometry reveals tradeoffs between productivity and diversity
Source: Front Microbiol. 2023 Jan 5;13:910390. doi: 10.3389/fmicb.2022.910390 (PMC9849913; doi:10.3389/fmicb.2022.910390)

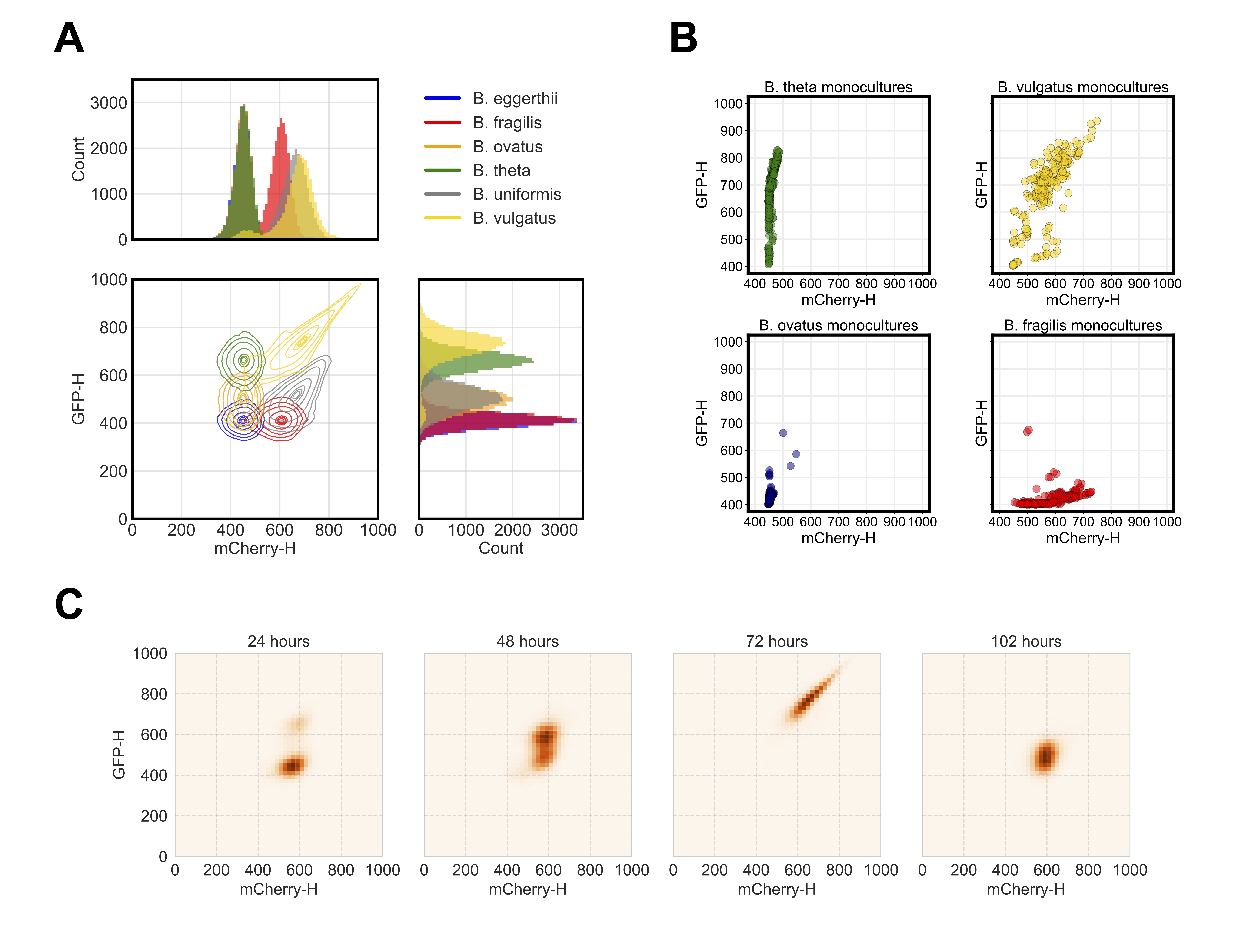

Supplement: SUPPLEMENTARY FIGURE 1 — Flow cytometry distinguishes multiple fluorescent Bacteroides species. (A) Flow cytometry analysis of events (total of 25,000 events per species) from six species pooled together post-processing. Species were grown overnight in rich media then washed and incubated in aerobic conditions for 6 hours prior to analysis with flow cytometry. Cellular events were characterized by height of their signals either in green (GFP-H) or red (mCherry-H) fluorescence channels. The two-dimensional panel shows contour lines corresponding to boundaries where there is a 95%, 90%, 80%, 50%, 20%, 10%, and 5% probability (from outermost to innermost line) for the position of cells in each cluster based on gaussian kernel density estimation. Side panels shows histograms for the actual count of events either in the red (top) or green (side) fluorescence channel. (B) Flow cytometry clusters in monoculture samples exhibited variation in their fluorescence intensities. Here, each data point is a cluster centroid which corresponds to the mean intensity of GFP and mCherry for all events in a cluster. Clusters correspond to monocultures sampled at four different time points (24, 48, 72, and 102 hours) and grown on 34 different media conditions used in this study. For each monoculture sample, we only show the primary cluster that corresponds to the cluster with the highest weight (as estimated by Gaussian Mixture Models). (C) Two-dimensional flow cytometry histograms for monoculture of B. vulgatus grown on minimal medium supplemented with fructose. Here, multiple clusters can correspond to cells from the same monoculture as seen for 24 hours and 48 hours, and centroids of these clusters can shift over time. [file Image_1.jpg]

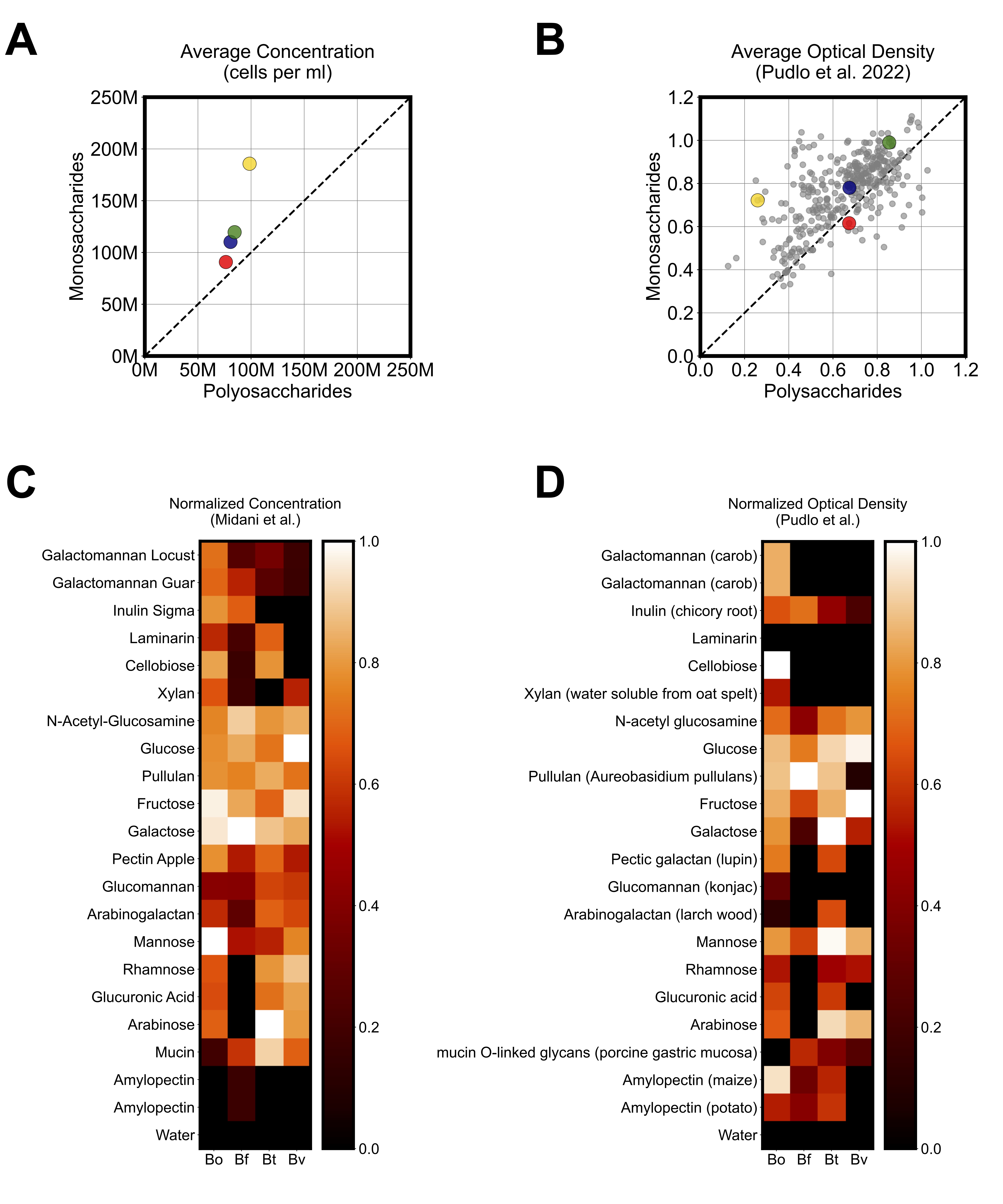

Supplement: SUPPLEMENTARY FIGURE 3 — Comparison of monoculture assays between flow cytometry-based measurements in this work and spectrophotometry-based measurements by Pudlo et al. (A) Scatter plot for the average species species productivity of Bacteroides isolates on 11 and 20 polysaccharides based on flow cytometric assay used in this study. Colors indicate species: B. ovatus (blue), B. fragilis (red), B. thetaiotaomicron (green), B. vulgatus (gold). (B) Scatter plot for the average species productivity of Bacteroides isolates on 15 monosaccharides and 30 polysaccharides based on spectrophotometric assays by Pudlo et al. Here, we color data points belonging to the parental type strains for the isolates used in this study. Circles that are above the dashed line correspond to isolates that had higher average growth on monosaccharides than on polysaccharides. Averages were computed only on substrates that supported the growth of each isolate. (C,D) Comparison of the results of the growth assays in this study and assays in Pudlo et al. (2022). Pudlo and colleagues displayed growth data in heatmaps after normalizing the optical density values for each species such that the growth on the substrate that provided maximum total growth for each strain was set to 1. To fairly compare growth data in our manuscript to the growth data in Pudlo et al. (2022), we applied a similar normalization scheme. Therefore, (C) displays maximum absolute abundances which were normalized for each species to a range of 0 to 1, and (D) displays optical density values which were normalized for each species to a range of 0 to 1. In all plots, “M” indicates values in the millions. [file Image_3.jpg]

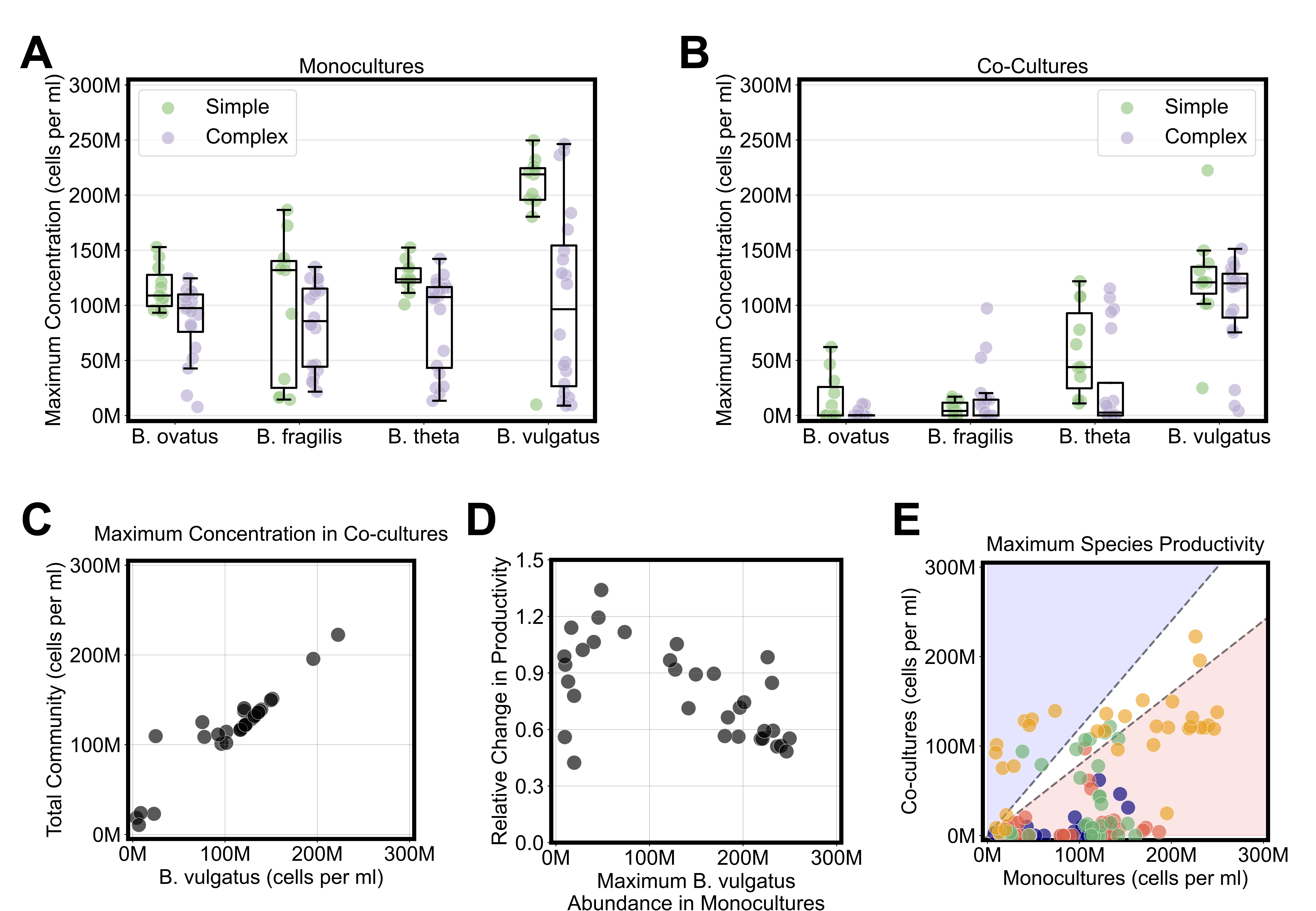

Supplement: SUPPLEMENTARY FIGURE 5 — Bacteroides vulgatus dominates co-cultures, impacts community productivity, and benefits from cross feeding. (A,B) The maximum abundances for each species split by substrate complexity in monocultures and co-cultures respectively. (C) Plot shows the maximum abundance of B. vulgatus in co-cultures against the maximum total abundance of the four-member community in co-cultures. (D) Plot shows the maximum abundance (cells per ml) of B. vulgatus in monocultures against the relative change in productivity, defined as the ratio of “maximum co-culture productivity” to “maximum monoculture productivity,” for each substrate. (E). Plot shows the maximum productivity (cells per ml) of each species in co-cultures vs. monocultures for all substrates. Shaded blue and red regions indicate growths in co-cultures that are at least 20% higher or lower than monocultures respectively. Colors indicate species: B. ovatus (blue), B. fragilis (red), B. thetaiotaomicron (green), and B. vulgatus (gold). [file Image_5.JPEG]
